# Acute hemodynamic effects of a multi-ingredient performance supplement on brachial artery vasodilation and blood flow volume following elbow flexion exercise in healthy young men

**DOI:** 10.1186/1550-2783-12-S1-P28

**Published:** 2015-09-21

**Authors:** Roxanne M Vogel, Jordan M Joy, Paul H Falcone, Matt M Mosman, Aaron C Tribby, Chad M Hughes, Jonathan D Griffin, Schyler B Tabor, Dylan J LeFever, Stephen B McCaughey, Michael P Kim, Jordan R Moon

**Affiliations:** 1 MusclePharm Sports Science Institute, Denver, CO, USA; 2 Department of Human Performance, Concordia University Chicago, River Forest, IL, USA; 3 Department of Movement Science, Grand Valley State University, Allendale, MI, USA; 4 Department of Biomedical Engineering, Widener University, Chester, PA, USA; 5 The Hospitality College, Johnson and Wales University, Denver, CO, USA; 6 Department of Human Performance and Sport, Metropolitan State University, Denver, CO, USA; 7 Department of Sports Exercise Science, United States Sports Academy, Daphne, AL, USA

## Background

Nutritional supplements have received attention for increasing blood flow to skeletal muscle during exercise. L-arginine is often used for its vasodilatory effects, and supplementation with nitrates has recently become more popular for the same reason. The purpose of the present study was to determine the acute hemodynamic effects of a multi-ingredient performance supplement (MIPS) containing arginine and nitrates as compared to placebo following resistance exercise in healthy young men.

## Methods

In a randomized double-blind, crossover, placebo-controlled design, 11 recreationally-active males (28.2 ± 5.0 y, 182.4 ± 5.7cm, 87.1 ± 10.3kg) ingested either 1 serving (14.5 g) of a MIPS (SUPP; Assault™, Musclepharm, Denver, CO) or a flavor-matched, visually identical placebo (PLA) and performed 3 sets of 15 arm curls at 30 minutes (30P) and 120 minutes (120P) post-supplementation. Brachial artery vessel diameter (VD) and blood flow volume (BFV) were measured via Doppler ultrasound at 0, 3, and 6 minutes post-exercise. Additionally, BP, HR, and BIA-determined extracellular water (ECW) and intracellular water (ICW) were assessed. Measurements taken following 30P and 120P were compared with both resting baseline (no treatment, no exercise) and active control (no treatment, exercise) values. Data were analyzed for all group, time, and group × time interactions using 2-way repeated-measures ANOVA. Alpha was predetermined at p < 0.05.

## Results

A significant (p < 0.05) group × time interaction was present for brachial artery VD, wherein SUPP increased to a greater extent than PLA at 0 minutes following 30P compared to both resting baseline (SUPP +0.09 ± 0.03cm; PLA +0.06 ± 0.03cm) and active control (SUPP +0.05 ± 0.04cm; PLA +0.02 ± 0.02cm) values. However, the increase in BFV at 0 minutes following 30P did not vary significantly between treatments from either resting baseline (p = 0.49) or active control (p = 0.27) values. No other variables had significant (p < 0.05) group × time interactions between any other time points.

## Conclusion

Acute supplementation with a multi-ingredient performance supplement containing arginine and nitrates may increase vasodilation synergistically with resistance exercise 30 minutes post-ingestion. However, it remains to be seen if increased vasodilation necessarily results in increased blood flow volume to working musculature.

